# Increase in Free Hepatic Venous Pressure Response to Beta-Blockers Predicts Variceal Bleeding in Cirrhotic Patients

**DOI:** 10.1155/2021/5587566

**Published:** 2021-04-26

**Authors:** Huiwen Guo, Jiangqiang Xiao, Yi Wang, Ming Zhang, Yuzheng Zhuge, Feng Zhang

**Affiliations:** Department of Gastroenterology, Affiliated Drum Tower Hospital of Nanjing University Medical School, Nanjing, Jiangsu, China

## Abstract

**Background and Aims:**

Nonselective beta-blockers (NSBBs) are the main drug to prevent portal hypertension. It could alter free hepatic venous pressure (FHVP); however, the significance is unknown. This prospective study was to explore the change of FHVP after use of NSBBs and its predictive value for gastroesophageal varices (GOV) bleeding in cirrhotic patients. *Patients and Methods*. Cirrhotic patients with medium-large GOV between September 2014 and January 2019 were enrolled. After initial hepatic venous pressure gradient (HVPG) measurement, patients received oral NSBBs. Seven days later, the secondary HVPG was examined to evaluate the FHVP alteration and hemodynamic response. The variceal bleeding between patients with FHVP increased and decreased/unchanged was compared.

**Results:**

A total of 74 patients were enrolled, and 62 patients completed the secondary HVPG measurement and was followed up. The cumulative bleeding rate was significantly higher in patients with FHVP increased ≥ 1.75 mmHg than those with FHVP decreased/unchanged (54.5% vs. 22.5%, *p* = 0.021), while there was no significant difference in bleeding between HVPG responders and nonresponders (32.6% vs. 37.5%, *p* = 0.520). For HVPG responders, variceal bleeding in patients with FHVP increased ≥ 1.75 mmHg was significantly more than that in patients with FHVP decreased/unchanged (57.9% vs. 28.6%, *p* = 0.041). Cox regression analysis showed that change of FHVP was an independent predictor of variceal bleeding.

**Conclusion:**

Increase ≥ 1.75 mmHg in FHVP responding to beta-blockers in cirrhotic patients with GOV indicates high risk of variceal bleeding. Besides HVPG response, change of FHVP should also be valued in hemodynamic evaluation to beta-blockers. This trial is registered with Chinese Clinical Trial Registry ChiCTR-IPR-17012836.

## 1. Introduction

Portal hypertension, one of the main clinical features of patients with decompensated cirrhosis, is a common critical and severe disease. Gastroesophageal varices (GOV) bleeding is the main cause of death in patients with portal hypertension, with a bleeding rate of 10%-15% per year and a 6-week mortality rate of 15%-25% [1, 2]. So far, nonselective beta-blockers (NSBBs) are the basic drug that can reduce portal pressure [3], which is the main means of primary prophylaxis of variceal bleeding and the cornerstone of secondary prevention [4]. However, not all patients can benefit from NSBBs. Because of no hemodynamic response, patients might still occur variceal bleeding.

Hepatic venous pressure gradient (HVPG), the difference between wedged hepatic venous pressure (WHVP) and free hepatic venous pressure (FHVP), is an effective way to evaluate the hemodynamic response of NSBBs. Both the consensus of Baveno VI [5] and the consensus of American Association for the Study of Liver Diseases 2016 [4] clearly pointed out and emphasized the role of HVPG in the risk stratification of portal hypertension and the predictive value of prognosis in cirrhotic patients. Therefore, HVPG is considered to be a good predictor of clinical decompensation of portal hypertension. However, it is reported that only about 50% patients receiving NSBBs can achieve hemodynamic response [6, 7].

Previous studies showed that in the secondary prevention of variceal bleeding, the rebleeding rate of HVPG responders is lower compared to nonresponders [8–10]. However, some hemodynamic responders still suffered GOV bleeding [11–13]. It seemed that HVPG response could not completely predict variceal bleeding, which indicated there might be other factors unrevealed. In clinical practice, we found that FHVP could be increased, unchanged, or decreased by measuring HVPG again after taking NSBBs. However, the specific significance of FHVP change is unknown, and to the best knowledge of us, there is no study reporting this at present.

Therefore, we conducted this study to explore the significance of FHVP change response to beta-blockers and its predictive value for GOV bleeding in patients with cirrhosis and portal hypertension.

## 2. Patients and Methods

### 2.1. Patients

This a prospective observational cohort study performed at the Department of Gastroenterology in the Affiliated Drum Tower Hospital of Nanjing University between September 2014 and January 2019. The inclusion criteria of the study was as follows: (1) 18 to 75 years old, (2) diagnosis of cirrhosis on the basis of clinical and imaging features or liver biopsy, (3) presence of medium-large GOV determined by a recent upper endoscopy procedure, (4) for secondary prevention, and (5) provision of written informed consent. The exclusion criteria was as follows: (1) contraindications to NSBBs (asthma, severe chronic obstructive lung disease, sinus bradycardia, II-III degree atrioventricular block, etc.); (2) taking drugs that affect the metabolism and absorption of NSBBs in the body at the same time, such as rifampicin and cimetidine; (3) using other drugs that reduce portal pressure; (4) concomitant malignant tumors and severe portal vein thrombosis; (5) severe heart, lung, liver, kidney dysfunction, or severe bleeding disorders such as thrombocytopenic purpura and hemophilia or local/systemic infections, hypothyroidism, Raynaud's syndrome, peripheral vascular disease, etc.; (6) patients with spontaneous portosystemic shunt; (7) women planning to become pregnant or breastfeeding; (8) other disease affecting life expectancy; (9) failure of HVPG measurement or initial HVPG value < 12 mmHg. The whole study was performed following the principles of the 1975 Declaration of Helsinki and was approved by the Ethics Committee of the Affiliated Drum Tower Hospital of Nanjing University.

### 2.2. Study Design

The HVPG measurement procedures were described as previous studies [14, 15]: in brief, a 7-F balloon-tipped catheter (Edwards Lifesciences, Irvine, California, USA) was guided into the right or middle hepatic vein to measure the WHVP and FHVP, for three times, and the HVPG was calculated. After the initial HVPG measurement, the patients received oral propranolol or carvedilol. Carvedilol was started at 6.25 mg every day and increased to 12.5 mg every day one week later. Propranolol was started at 10 mg twice a day and increased by 10 mg stepwise daily until up to 40 mg twice a day or until the target dose was achieved. Blood pressure and pulse were closely monitored to maintain systolic blood pressure > 90 mmHg and heart rate > 55 bpm. Next, HVPG measurement was performed seven days later. Finally, patients were divided into different groups according to the change of FHVP and HVPG response. According to the guidelines [5], HVPG response was defined as a decrease of HVPG ≥ 10% or less than 12 mmHg. Patients were all treated by NSBBs combined with endoscopic therapy. Endoscopic therapy was performed every 1-4 weeks until the eradication of varices.

### 2.3. Follow-Up

Patients' follow-up was performed by outpatient clinic and telephone calls to record the patient's condition and details of clinical events at months 1, 3, and 6 and every 6 months thereafter. First EGD review was performed 3-6 months after eradication and every 6-12 months thereafter. The follow-up was ended on October 2019. The primary endpoint was GOV bleeding. The secondary endpoint included transplant-free survival. The patient would be censored at the time if liver transplantation was performed or alive until the deadline. Survival time was calculated from the date of the initial HVPG procedure.

### 2.4. Statistical Analysis

SPSS version 25.0 (SPSS Inc., Chicago, Illinois, USA) software was used for all statistical analysis. Quantitative data conforming to normal distribution were presented as mean ± standard deviation, whereas data not conforming to normal distribution were represented by median (range), and classification variables were expressed as counts and percentages. Independent sample *t*-tests and chi-square tests were used to compare patients between different groups. A receiver operating characteristic (ROC) curve analysis was performed to calculate the sensitivity, specificity, and positive and negative predictive values. The FHVP value with the best specificity and sensitivity (Youden's index) was chosen to optimize the predictive ability of GOV bleeding. The cumulative probability of the remaining patients who did not exhibit GOV bleeding was evaluated via Kaplan-Meier curves. The univariate and multivariate Cox proportional hazards models were used to detect independent predictors of GOV bleeding. Statistical significance was established at *p* < 0.05.

## 3. Results

### 3.1. Patients' Baseline Characteristics

**Figure 1:**
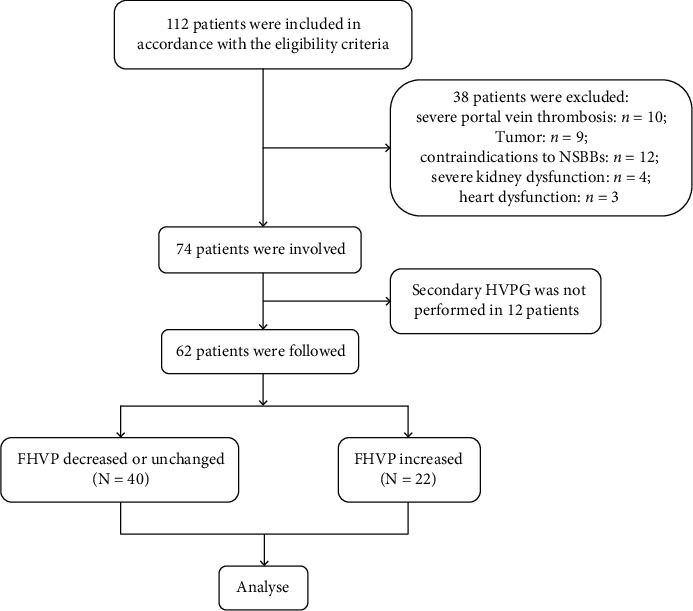
Flow chart of patients involved.

**Figure 2:**
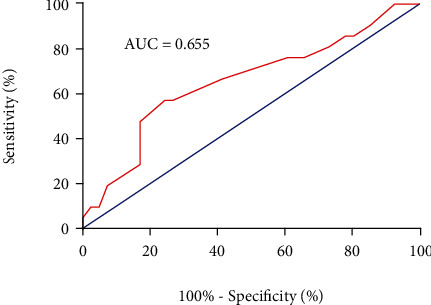
Receiver operating characteristic (ROC) curve of change of FHVP in predicting variceal bleeding.

During the study period, a total of 112 patients were enrolled in accordance with the inclusion criteria and 38 of them were excluded because of severe portal vein thrombosis (*n* = 10), tumor (*n* = 9), contraindications to NSBBs (*n* = 12), severe kidney dysfunction (*n* = 4), and heart dysfunction (*n* = 3). HVPG was measured successfully in all patients. During the follow-up, the second HVPG measurement was not performed in 12 patients. Finally, 62 patients were followed up and included in the analysis (Figure 1). The median follow-up time was 12.84 months (range from 0.06 to 49.00 months). 15 (24.2%) patients took carvedilol, and 47 (75.8%) patients took propranolol. All patients received NSBBs for secondary prophylaxis. The demographic characteristics, biochemical indicators, and imaging examinations of the patients were summarized in Table 1. ROC curve (Figure 2) and Youden's index were performed to optimize the predictive ability of GOV bleeding that the biggest Youden index was 0.327 and area under the receiver operating characteristic (AUROC) was 0.655 (0.505-0.805) (*p* = 0.047). The cut-off value of FHVP was increased 1.75 mmHg, and the sensitivity and specificity were 57.1% and 75.6%, respectively. According to the best cut-off value of FHVP change, we divided all patients into two groups of FHVP increased and FHVP decreased/unchanged. There were no significant differences in patients' demographics, liver disease characteristics, and clinical presentation between groups of FHVP decreased/unchanged and FHVP increased. Also, HVPG responders and nonresponders were comparable with respect to most features except prothrombin time (PT) and international standard ratio (INR) (Table 2).

### 3.2. Hemodynamic Response

**Figure 3:**
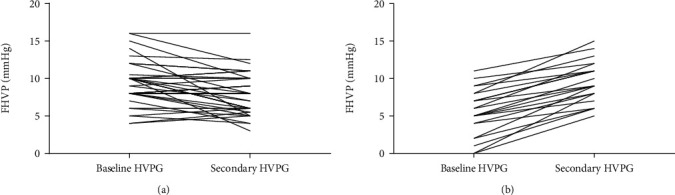
(a) Change of FHVP for patients with FHVP decreased or unchanged. (b) Change of FHVP for patients with FHVP increased.

The hemodynamic parameters of patients were summarized in Table 3. Baseline FHVP for patients with FHVP decreased/unchanged and FHVP increased were 9.29 ± 2.93 and 5.50 ± 3.13 (*p* < 0.001), and the secondary FHVP were 7.94 ± 2.77 and 9.55 ± 2.72 (*p* = 0.032), respectively (Figures 3(a) and 3(b)). Finally, 40 patients were distributed into group of FHVP decreased/unchanged and 22 patients were divided into group of FHVP increased. For HVPG nonresponders and responders, the HVPG decrease values were −0.09 ± 2.80 and 4.09 ± 2.24 (*p* < 0.001). All enrolled patients were divided into 46 HVPG responders and 16 nonresponders. Twenty-nine (72.5%) patients were considered to be hemodynamic responders in the FHVP decreased/unchanged group and seventeen (77.3%) patients in the FHVP increased group, while the difference was not significant (*p* = 0.769). Meanwhile, the change of FHVP was also not significant between HVPG responders and nonresponders (*p* = 0.670), indicating that the two groups were comparable.

### 3.3. Increase of FHVP Predicts Variceal Bleeding

**Figure 4:**
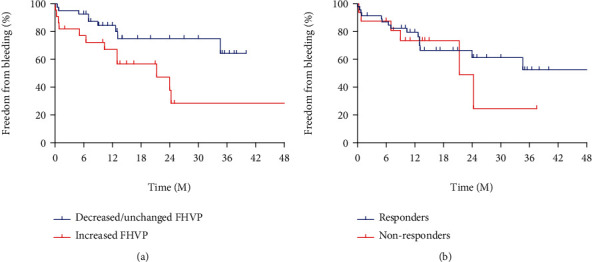
(a) Kaplan-Meier curve stratified by alteration of FHVP in predicting variceal bleeding. (b) Kaplan-Meier curve stratified by HVPG response in predicting variceal bleeding.

During the follow-up, 21 patients suffered bleeding, among them were 12 patients with FHVP increased and 9 with FHVP decreased/unchanged. All the bleeds were originated from GOV. Compared with the FHVP increased group, the cumulative variceal bleeding rate was significantly lower in the FHVP decreased/unchanged group (22.5% vs. 54.5%, *p* = 0.021, Figure 4(a)) and one patient with FHVP decreased/unchanged underwent liver transplantation due to uncontrolled variceal bleeding. However, the probability of bleeding in the group of HVPG responders was similar with the group of nonresponders (32.6% vs. 37.5%, *p* = 0.520, Figure 4(b)).

**Figure 5:**
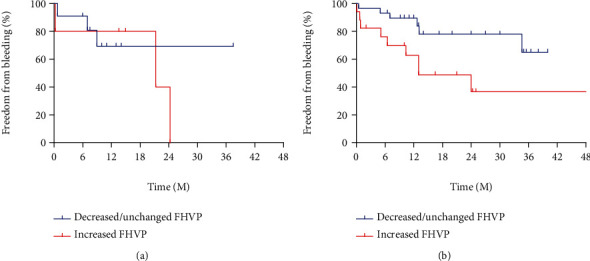
(a) Kaplan-Meier curve of nonresponders stratified by alteration of FHVP in predicting variceal bleeding. (b) Kaplan-Meier curve of responders stratified by alteration of FHVP in predicting variceal bleeding.

We also evaluated the cumulative bleeding rate of patients stratified by combination of alteration of FHVP and hemodynamic response. We divided all the patients into four subgroups. We found that in HVPG responders, the bleeding rate was significant lower in the patients with decreased/unchanged FHVP compared to those with increased FHVP. Specifically, in terms of nonresponders, there were no significant differences in the variceal bleeding rate between patients with FHVP decreased/unchanged and those with increased FHVP (27.3% vs. 60.0%, *p* = 0.613, Figure 5). However, for responders, bleeding rate of patients with increased FHVP was significantly higher than patients with FHVP decreased/unchanged (52.9% vs. 20.7%, *p* = 0.023, Figure 5(b)). 47 patients who took propranolol and 15 who took carvedilol were divided into two groups according to the change of FHVP, respectively. For patients administrated propranolol, the rate of variceal bleeding of those with FHVP increased was significantly higher than patients with FHVP decreased/unchanged (57.9% vs. 28.6%, *p* = 0.041), while in patients taking carvedilol, there was no significant difference in bleeding rate between the two groups (33.3% vs. 8.3%, *p* = 0.307). Compared with propranolol, carvedilol seemed to be more effective in reducing HVPG (3.21 ± 2.89 vs. 2.94 ± 3.06 mmHg, *p* = 0.766), but there was no significant difference. Also, no significant difference was observed in rate of hemodynamic response between carvedilol and propranolol (73.3% vs. 74.5%, *p* = 1.000).

### 3.4. Predicting Factors Associated with Variceal Bleeding

We took gender, age, total bilirubin, Child-Turcotte-Pugh (CTP) grade, ascites, change of FHVP, hemodynamic response, and baseline HVPG into univariate analysis and found that only the change of FHVP was significantly associated with the cumulative variceal bleeding rate. Furthermore, we included the FHVP into multivariate analysis and finally concluded that change of FHVP was an independent predictor of variceal bleeding (HR 2.692; 95% CI, 1.123-6.457, *p* = 0.026). Patients were significantly associated with higher bleeding rate if FHVP was increased (Table 4).

## 4. Discussion

In this study, we found that after receiving NSBBs, patients with FHVP decreased/unchanged had significantly lower cumulative variceal bleeding rate compared to those with FHVP increased (22.5% vs. 54.5%, *p* = 0.021). On the other hand, the variceal bleeding rate of HVPG responders was similar to that of nonresponders (32.6% vs. 37.5%, *p* = 0.520). In subgroup analysis, it was found that the bleeding rate of patients with FHVP increased was significantly higher than that of patients with FHVP decreased/unchanged (52.9% vs. 20.7%, *p* = 0.023) in HVPG responders. Through Cox regression analysis, change of FHVP is an independent predictor of high risk of variceal bleeding. To the best knowledge of us, the present study for the first time focused on FHVP change by the efficacy of NSBBs.

HVPG is the gold standard for the diagnosis of portal hypertension and the evaluation of drug efficacy [16], which has been proved to be an excellent predictor of prognosis in GOV bleeding, risk of liver decompensation, and liver failure after liver transplantation [17, 18]. A previous study involving 65 cirrhotic patients with acute variceal bleeding and early detection of HVPG after admission showed that the only variable associated with worse prognosis was an increase in HVPG, and an initial HVPG ≥ 20 mmHg was associated with a decrease in short-term or long-term survival [19]. Therefore, the measurement of HVPG is of great significance in patients with portal hypertension.

NSBBs are recommended as the first-line treatment of primary prevention and the cornerstone of secondary prevention of GOV bleeding in cirrhosis [4, 5]. NSBBs can reduce portal vein pressure, decrease the incidence of variceal bleeding and hepatic encephalopathy, and improve survival [20]. Propranolol has been shown to reduce the risk of rebleeding (34%) and mortality (26%) in patients with a history of variceal bleeding [21]. For patients who received NSBBs combined with sequential endoscopic therapy for secondary prevention, the use of NSBBs is the only independent predictor of reduced risk of varices recurrence [22]. Unfortunately, NSBBs are not effective for all patients, and only about half of the patients respond to the treatment [6].

A large number of studies have shown that when HVPG responds to NSBBs, the risk of GOV bleeding is significantly reduced [9, 10]. HVPG provides valuable prognostic information for monitoring the effectiveness of drug therapy [23]. HVPG is usually defined as the difference between WHVP and FHVP. In our clinical practice, we found that FHVP could be increased, unchanged, or decreased when patients retested HVPG after taking NSBBs, but the specific significance has not been reported so far; therefore, we conducted this prospective study and found that the clinical prognosis of patients with FHVP increased was different from that of patients with FHVP decreased/unchanged.

In the present study, we divided the 62 patients into HVPG responders and nonresponders (HVPG decrease ≥ 10% of the baseline or to ≤12 mmHg as “responders”), among which 15 patients took carvedilol and 47 patients took propranolol, and a review showed that although carvedilol was more effective in reducing HVPG, carvedilol had no significant beneficial or harmful effects on mortality, upper gastrointestinal bleeding, and severe or nonserious adverse events compared to traditional nonselective beta-blockers (propranolol or nadolol). The results showed that there was no significant difference in the rate of variceal bleeding between HVPG responders and nonresponders. This result indicated that besides HVPG, other factors predicting bleeding should be explored. A meta-analysis of ten studies enrolled 595 cirrhotic patients showed that the relative risk of bleeding and liver-related mortality in patients with HVPG response was lower than those with no response to HVPG [24], which was not consistent with our results. The reason may be that we included a small number of patients, with 46 responders and only 16 nonresponders.

During the follow-up, we found that the rate of GOV bleeding in patients with FHVP increased was higher than that in patients with FHVP decreased/unchanged. Among the HVPG responders, variceal bleeding rate of the patients with FHVP increased was also significantly higher than that of patients with FHVP decreased/unchanged. 77.3% patients (17/22) with increased FHVP were evaluated as HVPG responders, while 72.5% patients (29/40) with FHVP decreased/unchanged were evaluated as HVPG responders, indicating that there was no significant correlation between HVPG response and FHVP change (*p* = 0.769). Although NSBBs play an important role in the prevention of variceal rebleeding, there are still a series of problems, such as NSBB-related side effects and the use of NSBBs in cirrhotic patients with spontaneous bacterial peritonitis, and refractory ascites is still controversial [25]. In addition, studies have shown that the use of NSBBs is a risk factor for portal vein thrombosis [26–28]. In our study, two patients who took propranolol decreased from 80 mg/d to 60 mg/d due to decreased heart rate, and no obvious adverse reactions were found in other patients. During the follow-up, no new portal vein thrombosis was found by conventional Doppler ultrasound. Therefore, not only HVPG but also FHVP should be paid attention to when evaluating hemodynamic response. When FHVP change is over 1.75 mmHg, a more aggressive treatment should be sought as soon as possible.

Some limitations of this study should be noted. First, although the study was a prospective cohort study, it was conducted in a single center with a small sample size. Second, there is a lack of detailed records of new decompensated events during long-term follow-up. Third, the cohort patients took different NSBBs involving propranolol and carvedilol. Although carvedilol was more effective than propranolol in reducing the HVPG, but there was no significant difference in variceal bleeding [29]. Therefore, more prospective multicenter clinical trials with rigorous study design, larger sample size, and comprehensive follow-up may be required to validate our results.

## 5. Conclusion

Increase ≥ 1.75 mmHg in FHVP responding to beta-blockers in cirrhotic patients with GOV indicates high risk of variceal bleeding. Besides HVPG response, change of FHVP should also be valued in hemodynamic evaluation to beta-blockers.

## Figures and Tables

**Table 1:** Patients' demographics, liver disease characteristics, clinical presentation, and hemodynamic parameters.

	Total
Mean age(years)	54.53 ± 11.32
Gender male/female	34/28
Etiology virus/alcoholic/PBC^1^/others	37/7/5/13
PLT^2^ (×10^9^/L)	71.45 ± 51.69
TBil^3^ (*μ*mol/L)	18.61 ± 11.64
Creatinine (*μ*mol/L)	61.14 ± 18.41
Albumin (g/L)	35.44 ± 4.42
PT^4^ (s)	14.39 ± 2.38
INR^5^	1.25 ± 0.19
CTP^6^ scores	7 (5-9)
CTP stage A/B/C	31/31/0
Ascites	
No/mild/moderate/massive	24/22/15/1
Splenectomy yes/no	3/59
Baseline WHVP^7^ (mmHg)	23.80 ± 4.11
Baseline FHVP^8^ (mmHg)	7.94 ± 3.49
Baseline HVPG^9^ (mmHg)	15.85 ± 3.04
Baseline RAP^10^ (mmHg)	5.48 ± 2.80
Secondary WHVP (mmHg)	21.34 ± 4.10
Secondary FHVP (mmHg)	8.51 ± 2.84
Secondary HVPG (mmHg)	12.83 ± 3.28
Secondary RAP (mmHg)	6.66 ± 3.06
WHVP decrease value (mmHg)	2.46 ± 4.18
WHVP decrease percentage (%)	8.99 ± 18.02
FHVP increase value (mmHg)	0.56 ± 3.39
FHVP increase percentage (%)	28.16 ± 88.46
HVPG decrease value (mmHg)	3.01 ± 3.00
HVPG decrease percentage (%)	18.37 ± 18.66
Median follow-up time (m)	12.84 (0.06-49.00)

^1^PBC: primary biliary cirrhosis; ^2^PLT: platelets; ^3^TBil: total bilirubin; ^4^PT: prothrombin time; ^5^INR: international standard ratio; ^6^CTP: Child-Turcotte-Pugh; ^7^WHVP: wedged hepatic vein pressure; ^8^FHVP: free hepatic vein pressure; ^9^HVPG: hepatic venous pressure gradient; ^10^RAP: right atrial pressure. Ascites: (1) mild: patients generally have abdominal distension, the ascites can only be detectable by an ultrasound examination, and the depth is <3 cm. (2) Moderate: the patient often has moderate and symmetrical abdominal distension, and the depth is 3–10 cm. (3) Massive: the patient has a significant bloating. The ascites detected by ultrasound occupy the entire abdominal cavity, and the depth is >10 cm.

**Table 2:** Patients' demographics, liver disease characteristics, and clinical presentation (means ± standard deviation).

	FHVP^7^ decreased or unchanged (*N* = 40)	FHVP increased (*N* = 22)	*p* value	Responders (*N* = 46)	Nonresponders (*N* = 16)	*p* value
Mean age (years)	55.50 ± 10.61	52.77 ± 12.58	0.369	54.00 ± 11.44	56.06 ± 11.22	0.535
Gender male/female	19/21	15/7	0.182	27/19	7/9	0.386
Etiology virus/alcoholic/PBC^1^/others	21/5/3/11	16/2/2/2	0.326	30/5/3/8	7/2/2/5	0.465
PLT^2^ (×10^9^/L)	67.40 ± 40.09	78.82 ± 68.46	0.410	74.43 ± 58.17	62.88 ± 24.65	0.446
TBil^3^ (*μ*mol/L)	16.40 ± 8.38	22.63 ± 15.39	0.089	19.16 ± 10.65	17.02 ± 14.40	0.531
Creatinine (*μ*mol/L)	60.55 ± 19.56	62.23 ± 16.48	0.734	60.84 ± 19.65	62.03 ± 14.75	0.825
Albumin (g/L)	34.74 ± 4.03	36.71 ± 4.89	0.094	35.18 ± 4.69	36.16 ± 3.56	0.450
PT^4^ (s)	14.51 ± 2.67	14.17 ± 1.75	0.603	14.80 ± 2.52	13.19 ± 1.39	0.018
INR^5^	1.25 ± 0.22	1.23 ± 0.15	0.661	1.28 ± 0.20	1.14 ± 0.12	0.021
CTP^6^ scores	7 (5-9)	6.5 (5-8)	0.799	7 (5-9)	6 (5-8)	0.117
CTP stage A/B/C	20/20/0	11/11/0	1.000	20/26/0	11/5/0	0.146
Ascites^8^						
No/mild/moderate/massive	15/12/12/1	9/10/3/0	0.374	16/17/12/1	8/5/3/0	0.698
Splenectomy yes/no	1/39	2/20	0.285	3/43	0/16	0.562
Median follow-up time (m)	11.50	13.00	0.740	13.00	10.50	0.331

^1^PBC: primary biliary cirrhosis; ^2^PLT: platelets; ^3^TBil: total bilirubin; ^4^PT: prothrombin time; ^5^INR: international standard ratio; ^6^CTP: Child-Turcotte-Pugh; ^7^FHVP: free hepatic vein pressure; ^8^ascites: (1) mild: patients generally have abdominal distension, the ascites can only be detectable by an ultrasound examination, and the depth is <3 cm. (2) Moderate: the patient often has moderate and symmetrical abdominal distension, and the depth is 3–10 cm. (3) Massive: the patient has a significant bloating. The ascites detected by ultrasound occupy the entire abdominal cavity, and the depth is >10 cm.

**Table 3:** Hemodynamic parameters of patients (means ± standard deviation).

	FHVP decreased or unchanged (*N* = 40)	FHVP increased (*N* = 22)	*p* value	Responders (*N* = 46)	Nonresponders (*N* = 16)	*p* value
Baseline WHVP^1^ (mmHg)	24.53 ± 4.10	22.46 ± 2.86	0.057	23.77 ± 3.95	23.86 ± 4.68	0.942
Baseline FHVP^2^ (mmHg)	9.29 ± 2.93	5.50 ± 3.13	<0.001	7.84 ± 3.46	8.25 ± 3.68	0.687
Baseline HVPG^3^ (mmHg)	15.24 ± 2.97	16.96 ± 2.93	0.032	15.94 ± 3.18	15.61 ± 2.68	0.716
Baseline RAP^4^ (mmHg)	6.23 ± 2.50	4.14 ± 2.87	0.004	5.44 ± 2.83	5.63 ± 2.80	0.817
Secondary WHVP (mmHg)	20.47 ± 3.73	22.91 ± 4.35	0.024	20.34 ± 3.63	24.21 ± 4.09	0.001
Secondary FHVP (mmHg)	7.94 ± 2.77	9.55 ± 2.72	0.032	8.51 ± 2.74	8.50 ± 3.18	0.990
Secondary HVPG (mmHg)	12.54 ± 3.17	13.36 ± 3.48	0.348	11.83 ± 2.90	15.71 ± 2.55	<0.001
Secondary RAP (mmHg)	5.88 ± 2.83	8.07 ± 3.02	0.006	6.54 ± 2.90	7.00 ± 3.54	0.611
WHVP decrease value (mmHg)	4.06 ± 3.20	−0.44 ± 4.25	<0.001	3.44 ± 3.52	−0.34 ± 4.75	0.001
WHVP decrease percentage (%)	15.98 ± 11.29	−3.71 ± 21.08	<0.001	13.61 ± 13.28	−4.29 ± 23.19	0.009
FHVP increase value (mmHg)	−1.35 ± 2.39	4.05 ± 1.76	<0.001	0.67 ± 3.04	0.25 ± 4.34	0.670
FHVP increase percentage (%)	−11.64 ± 23.74	100.54 ± 114.80	<0.001	29.70 ± 85.94	23.73 ± 98.17	0.818
HVPG decrease value (mmHg)	2.68 ± 2.26	3.60 ± 4.01	0.251	4.09 ± 2.24	−0.09 ± 2.80	<0.001
HVPG decrease percentage (%)	17.80 ± 14.37	19.40 ± 25.03	0.750	25.46 ± 11.68	−2.02 ± 20.23	<0.001

^1^WHVP: wedged hepatic vein pressure; ^2^FHVP: free hepatic vein pressure; ^3^HVPG: hepatic venous pressure gradient; ^4^RAP: right atrial pressure.

**Table 4:** Univariate and multivariate analyses for factors associated with variceal bleeding.

	Univariate analysis		Multivariate analysis	
Variable	Hazard ratio (95% CI)	*p* value	Hazard ratio (95% CI)	*p* value
Age	0.994 (0.957-1.031)	0.734		
Gender (male vs. female)	0.963 (0.404-2.295)	0.932		
CTP^1^ grade				
A	Reference			
B	0.842 (0.352-2.011)	0.698		
Ascites				
No	Reference			
Mild	2693.596 (0-4.810*E*+103)	0.947		
Moderate	3194.624 (0-5.704*E*+103)	0.945		
Massive	3547.758 (0-6.337*E*+103)	0.945		
TBil^2^	1.012 (0.983-1.043)	0.417		
FHVP^3^ group				
FHVP decreased or unchanged	Reference		Reference	
FHVP increased	2.692 (1.123-6.457)	0.026	2.692 (1.123-6.457)	0.026
Hemodynamic response group				
Responders	Reference			
Nonresponders	0.733 (0.283-1.900)	0.523		
Baseline HVPG^4^	1.071 (0.927-1.238)	0.353		

^1^CTP: Child-Turcotte-Pugh; ^2^TBil: total bilirubin; ^3^FHVP: free hepatic vein pressure; ^4^HVPG: hepatic venous pressure gradient.

## Data Availability

All data are contained in the manuscript.
